# Micellization-induced amplified fluorescence response for highly sensitive detection of heparin in serum

**DOI:** 10.1038/s41598-020-66360-8

**Published:** 2020-06-10

**Authors:** Yeon Jin Jang, Boyun Kim, Euijin Roh, Hyunuk Kim, Seoung Ho Lee

**Affiliations:** 1 Department of Chemistry, Daegu University, Gyeongsan, 38453 Republic of Korea; 2 Institute of Natural Sciences, Daegu University, Gyeongsan, 38453 Republic of Korea; 3 Energy Materials Laboratory, Korea Institute of Energy Research, 152 Gajeong-ro, Yuseong-gu Daejeon, 34129 Republic of Korea

**Keywords:** Single-molecule fluorescence, Self-assembly

## Abstract

Fluorescence-based assays should be feasible in aqueous media for effectively detecting the biological factors. However, numerous sensors have limited signal transductions and low fluorescence quantum yields due to the ingerently reduced excited state energy of fluorophores in aqueous solution, which reduces their sensitivity. This necessitates a smart sensing approach with an amplified fluorescence response for analytes in aqueous solution. Herein, a new building block which self-assembles in aqueous media, giving a micellar sturcuture with the hydrophobic π-extended conjugated system at the core and hydrophilic groups at the periphery, was devised for the first time. We demonstrated that the aggregated fluorophores in a micelle induce amplified fluorescence quenching, in which the excited electron efficiently migrates through π-extended conjugated system in a micelle, as in a polymeric system. Such feature differentiates this sensing approach from the numerous fluorescence-based tools previously developed for sensitive detection. This new system exhibited highly sensitive signal transduction for specific analytes even under actual bioanalytical conditions.

## Introduction

Fluorescence techniques for efficient detection of chemical and biochemical analytes have been actively investigated because the intrinsic and extrinsic photophysics associated with the excited state of fluorophores provides a wealth of information about the structures, molecular behaviours, and binding interaction between the fluorescence matrix and analytes^1^. Functionalized fluorophores, the photophysical properties of which can be readily influenced by a variety of environmental factors, including solvent polarity, pH, and concentration, enable highly efficient fluoregenic detection with high sensitivity by controlling the photon in the excited states^1–3^. These probes must meet the requirements for efficient signal transduction and high luminescent efficiency, even in highly polar solvents or biological conditions, but most of the current probes rely on platforms such as polymers and metal-based nanoparticles as sensing tools, which exhibit relatively superior photophysical properties^4–6^. Nevertheless, the inherent sensing complexity induced by the lack of reproducibility in the synthesis of polymers and metal-based nanoparticles and a strong tendency towards disordered solidification between materials have increased the demand for alternative sensing approaches^7^.

Micelle-based probes have been considered a promising candidate as potential chemical- and bio-sensors due to their structural flexibility and versatility, along with the possibility of adjusting the parameters in a controlled-manner^8^. The micelles’ amphiphilic features, hydrophobic core and ionic surface allow two different molecular communications with lipophilic or oppositely charged species. In particular, most micelle-based sensors are able to signal the response of analyte detection by introducing an external fluorophore as an indicative marker (Supplementary Fig. 1)^9^. These structural and systemic features provide a very efficient sensing platform to a variety of chemical- and biomolecules, resulting in high selectivity and sensitivity^10^. Over the past decade, various micelle monomers and adaptable fluorophores have been developed and systematized to enhance the efficiency of the micelle-based fluorescence sensors^11–14^. Although numerous studies have led to a great interest in the development of new functional micelle-based sensors, novel domains enabling more efficient sensing transduction in the micellar field are still required for developing chemosensors with extremely high sensitivity^11^.

Anions present an important goal for bioanalytical chemistry because of their importance in various biochemical processes^15^. For example, heparin (HP) plays an important role in various physiological and pathological processes, such as cell growth, immune response, and regulation of blood coagulation^16–18^. Especially, it is used as an anticoagulant for medical purpose^18,19^. The abuse of HP has a side effect of usually causing hemorrhage, but in serious cases, it can lead to an increased risk of HP-induced thrombocytopenia^20^. Accordingly, detecting variations of HP levels in the bloodstream is not only important in the treatment of anticoagulant or HP antagonists, but is also a critical way to determine diagnoses associated with low blood platelets. HP assay must have low detection limits and should be performed in bioanalytical condition. Although numerous fluorescence-based assays have been developed and validated for monitoring the HP levels in aqueous media, their sensitivity is generally limited to micromolar or submicromolar ranges^21,22^. Therefore, it can be problematic to measure the ultralow HP concentrations in bioanalytical condition.

Herein, we report a novel fluorescent sensing approach that amplifies signal transduction based on interfluorophore exciton migration within a self-assembled conjugated micelle (CM). The correlation between the structural and photophysical properties of the CM provides insight into how its structural features induce amplified signal transduction, which is distinguished from common micelle-based sensors requiring an external fluorophore. As shown in Fig. 1a,b, the amphiphilic monomer 1, consisting of a hydrophobic alkylated coumarin unit and an ammonium moiety that is a water-solubilizing group, forms a micellar structure with the hydrophobic π-extended conjugated system in the inner part and hydrophilic functionality at the periphery, where the aggregated coumarin units at the core serve as a conduit for energy and electron transport. This new system affords exceedingly sensitive detection of selected analytes via amplified fluorescence quenching in biological media.

## Results and Discussion

**Figure 1:**
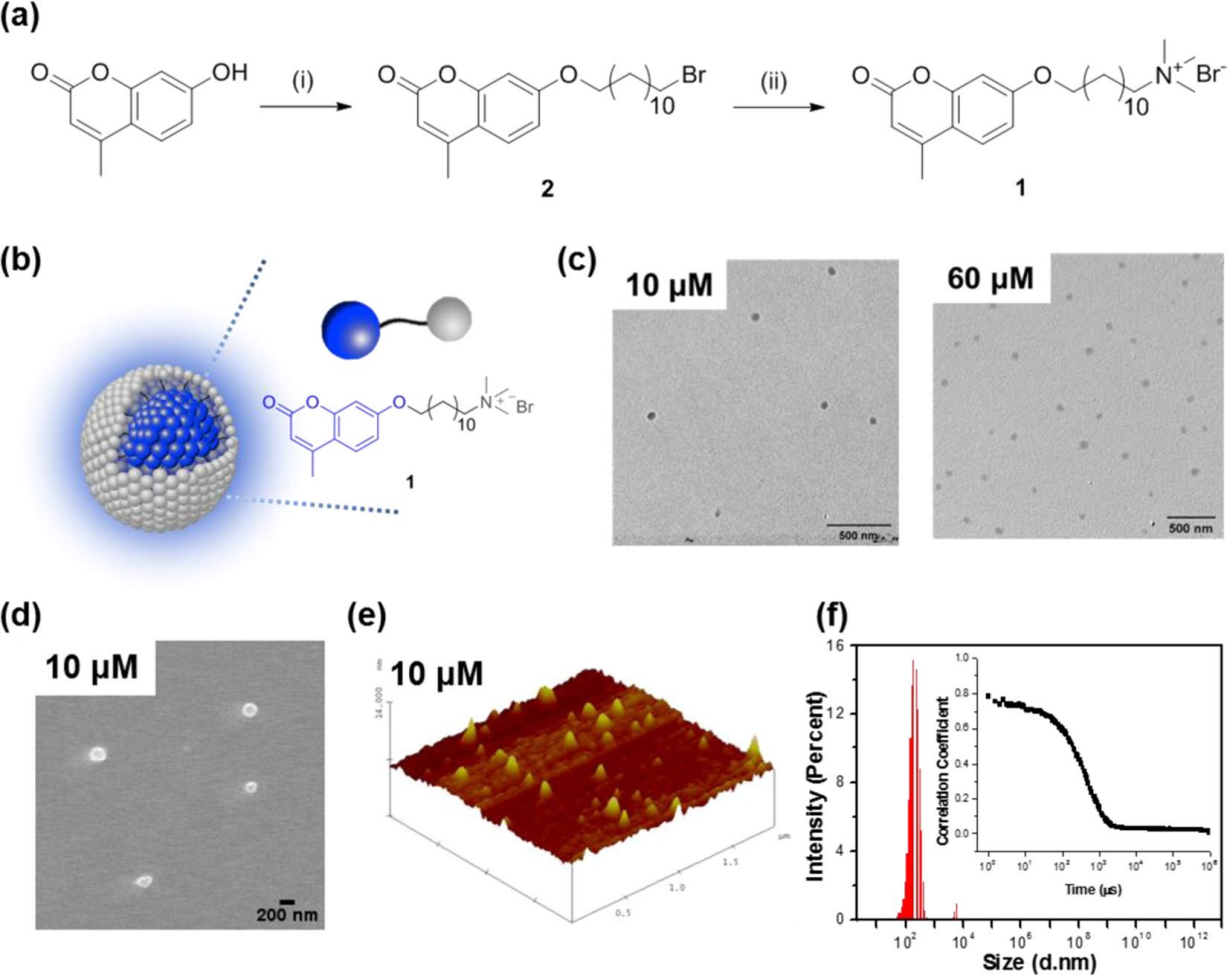
Synthetic procedure of 1 and its structural characterization. (**a**) Reagents and conditions: (i) 1,12-Dibromododecane, K_2_CO_3_, CH_3_CN, 80 °C, 75% yield; (ii) trimethylamine, THF, room temperature, 95% yield. (**b**) structure of micellized **1**. (**c**) HAADF-STEM images (scale bars, 500 nm) of micellized **1**. (**d**) FE-SEM image of **1** (1.0 × 10^-5^ M). (**e**) AFM image of **1** (1.0 × 10^-5^ M). (**f**) volume-based distribution of the hydrodynamic diameter and inset is correlation function obtained from dynamic light scattering (DLS) of **1** (60 × 10^−6^ M) in H_2_O.

### Structural studies

The synthetic pathway for amphiphilic building block **1** is described in Fig. 1a. The micellization of **1** was determined by high-angle annular dark-field scanning transmission electron microscopy (HAADF-STEM), field emission scanning electron microscopy (FE-SEM), Atomic force microscopy (AFM), and dynamic light scattering (DLS) studies in aqueous media. Figure 1c and Supplementary Fig. 2 present TEM images of **1** that was deposited on copper grids from 10 × 10^−6^ M and 60 × 10^−6^ M in aqueous solution, respectively. The sample exhibits micellar agglomerates (dark-contrast) with an average grain size of approximately 70 nm. This observation is consistent with FE-SEM and AFM results shown in Fig. 1d,e, where both FE-SEM and three-dimensional AFM images of **1** represent micelle-like particles with an average size of 178 nm and 123 nm, respectively. The DLS result also shows that a solution state of **1** represents an average size of 147 nm with the distribution profile showing a maximum size of approximately 190 nm (Fig. 1f). As shown in inset of Fig. 1f, the correlation of the signal gradually decays, which suggests that the micellized **1** represents a relatively large composite particle. Although TEM and DLS results exhibit different average sizes, this study shows that the size of micelles was not concentration dependent either in the solid state or the liquid state (Supplementary Fig. 2 and 3). On the other hand, neither TEM nor DLS results were obtained in methanol, which is a relatively good solvent for **1**. The micellization of **1** in aqueous media is attributed to intermolecular hydrophobic interaction between the alkylated coumarin units against the hydrophilic nature of water, and thus the aggregated coumarins are located inside the micelle where they can form a structure capable of photophysical communicating with each other.

### Photophysical properties

**Figure 2:**
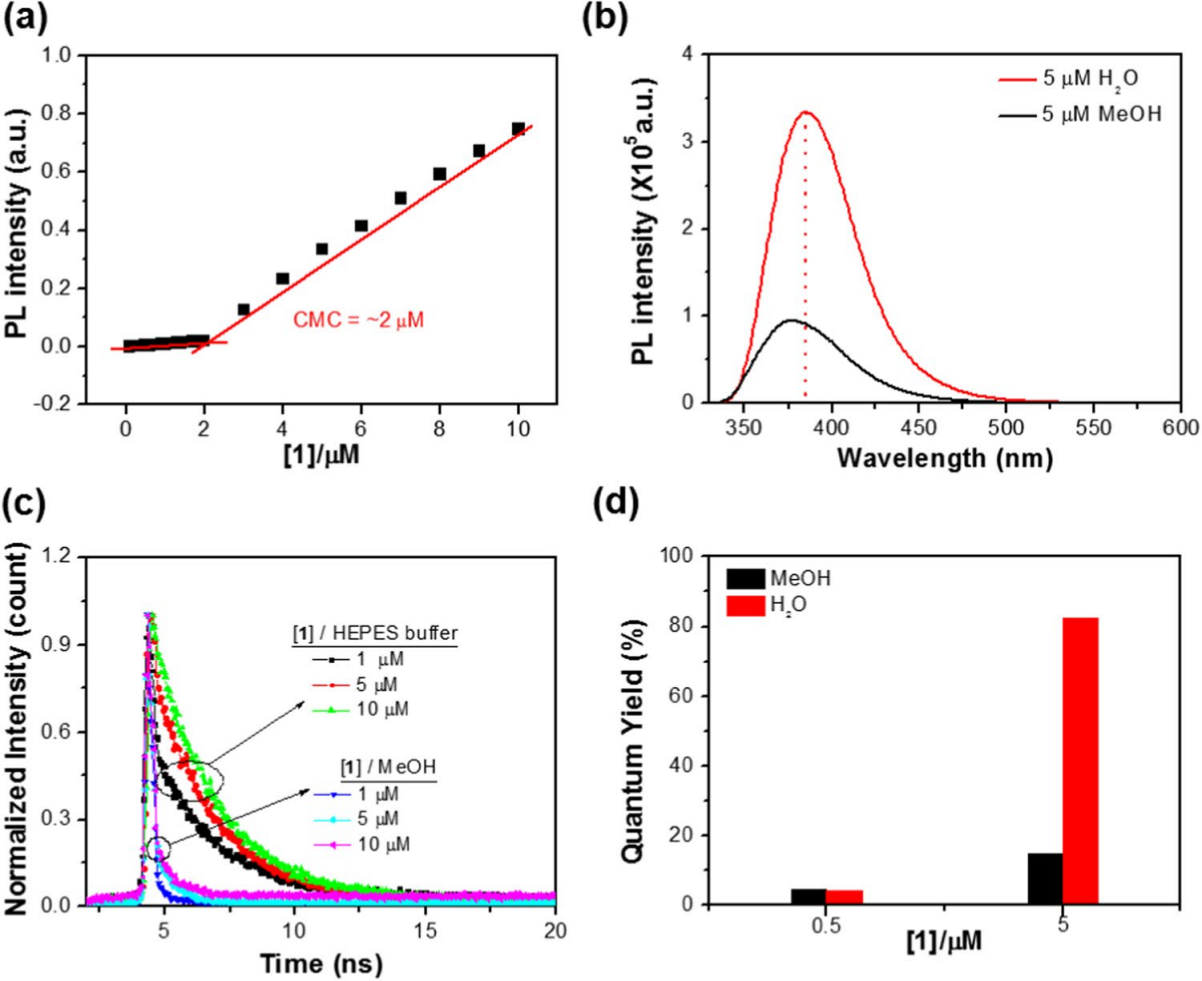
Correlation between structural and spectral behaviours of 1. (**a**) critical micelle concentration (CMC) determined by the change in fluorescence intensity as a function of the concentration. (**b**) fluorescence emission spectra of **1** (5.0 × 10^−6^ M) in MeOH and H_2_O. excitation at 320 nm. (**c**) time-resolved spectroscopic study below (1.0 × 10^−6^ M) and above (5.0 × 10^−6^ M and 1.0 × 10^−5^ M) the CMC of **1** in MeOH and 10 mM HEPES buffer solution at pH 7.4. (**d**) fluorescence quantum yields below (0.5 × 10^−6^ M) and above (5.0 × 10^−6^ M) the CMC points of **1** in MeOH and H_2_O. 9,10-diphenylanthracene in cyclohexane as the standard, Φ_FL_ = 0.95.

The aggregation of coumarin moieties in the CM was further investigated by UV/Vis and fluorescence spectroscopic studies in methanol and aqueous solution. Figure 2a and Supplementary Fig. 4a present the changes in the fluorescence emission intensity of **1** as a function of its concentration in aqueous solution. The fluorescence intensity was drastically increased from approximately 2.0 × 10^−6^ M, suggesting that the micelle formation begins at this concentration, i.e., the critical micelle concentration (CMC) is approximately 2.0 × 10^−6^ M. In methanol, however, the fluorescence intensity was proportionally changed with increasing concentration of **1**, implying that little or no aggregation is expected in this condition (Supplementary Fig. 4b). As shown in Fig. 2b, the fluorescence emission spectrum of micellized **1** (5.0 × 10^−6^ M) above the CMC in aqueous solution exhibits the red-shifted (7 nm) and increased intensity of the peak maximum (385 nm) compared to that in methanol. To explore this red-shift of micellized **1** in fluorescence emission spectra, its emission properties were studied in the mixed solvent systems of MeOH-H_2_O and/or THF-H_2_O with varied volumetric fractions of water (*f*_w_) (Supplementary Figs. 5 and 6). As the water is added to the methanol solution, the fluorescence spectra of **1** (5.0 × 10^−6^ M) was slightly changed (λ_max_ = 380 nm) until the *f*_w_ reached 30% and the fluorescence emission was gradually red-shifted with the *f*_w_ = 80%. Upon the further addition of water to methanol, the maximum wavelength of the spectra was retained at λ_max_ = 385 nm. The fluorescence intensity changes also showed similar features with the profile of the wavelength changes. This phenomenon was more pronounced in THF-H_2_O system, where THF is more ‘good solvent’ than methanol (Supplementary Fig. 6). This observation clearly indicates that the red-shift in the fluorescence emission spectra is more dependent on the aggregation issue of **1** rather than solvent polarity. Nevertheless, no change in fluorescence excitation spectrum of **1** (5.0 × 10^−6^ M) between methanol and aqueous solution was observed (Supplementary Fig. 7a). Based on above observations, it is likely that the red-shifted emission in aqueous solution is presumably due to stabilization of the fluorophores’ excited state relative to their aggregation. In addition, there was no remarkable change in UV/Vis absorption of **1** between monomeric state (below the CMC) and micellized state (above the CMC) in aqueous solution (Supplementary Fig. 7c). For this observation, we believe that the hydrophobic moieties of **1** are aggregated in the ground state and may not be in a favorable configuration for π-π interaction between aromatic units. As a result, the emission intensity enhancement in aqueous solution may be a consequence of a restriction in the conformational flexibility that would otherwise lead to nonradiative decay through rotatory motion of non-aggregated **1**^23^. In addition, the structural characteristic of micellized **1** can reduce the water contact of luminescence units, thereby inhibiting the nonradiative decay caused by contact with a high polar solvent^24^.

To elucidate intermolecular interactions in these excited states, time-correlated single photon counting (TCSPC) study was conducted for **1** in methanol and HEPES (4-(2-hydroxyethyl)piperazine-1-ethanesulfonic acid) buffer solution (0.01 M, pH 7.4). The fluorescence lifetimes (*τ*_*i*_) and their relative amplitude contributions (RA%) to the overall decays are described in Fig. 2c and Supplementary Table 1. The data show that **1** (1.0 × 10^−6^ M and 5.0 × 10^−6^ M) below and above the CMC points in methanol yields relatively fast decay (*τ*_1_ = 0.21 and 0.26 ns) that predominant contributes to over >95% of the overall amplitude and that the decay dynamics is relatively independent of its concentration. On the other hand, in aqueous solution, **1** (1.0 × 10^−6^ M) below the CMC point features nonlinear decay behaviour with average lifetime (*τ*_av._= 1.96 ns) comprised of two emissive pathways: 25% of short-lived decay (*τ*_1_ = 0.25 ns) and 75% of long-lived one (*τ*_2_ = 2.53 ns). This fast component showed a decreased contribution (*τ*_1_ = 0.22 ns, 3%; *τ*_2_ = 2.45 ns, 97%) above the CMC point, increasing the average lifetime to *τ*_av._= 2.40 ns. The results clearly demonstrate that the emission from this micellized **1** arise from a singlet state with considerable charge transfer character, presumably due to the long-live charge separation state of aggregated fluorophores in the excited state. Thus, based on the UV/Vis absorption/fluorescence and TCSPC studies, it is considered that such charge transfer character is generated by complex formation of a coumarin moiety with a excited stated coumarin moiety (coumarin + *coumarin) in the excited state because the decay dynamics was changed dependent on the concentration of the probe in the same solvent.

The fluorescence quantum yields for **1** were also measured below and above the CMC points in methanol and aqueous solution, respectively (Fig. 2d and Supplementary Fig. 8). In spite of different concentrations of **1**, relatively low fluorescence quantum yields were observed in methanol (Φ_FL_ = 4.8%/MeOH ([**1**] = 5.0 × 10^−7^ M), and Φ_FL_ = 15%/MeOH ([**1**] = 5.0 × 10^−6^ M). As expected, in aqueous solution, the fluorescence quantum yield below the CMC point was similar to that of methanol, but was drastically increased above the CMC point (Φ_FL_ = 4.2%/H_2_O ([**1**] = 5.0 × 10^−7^ M), Φ_FL_ = 74%/H_2_O ([**1**] = 5.0 × 10^−6^ M)). The slight increase in fluorescence quantum yield of **1** above CMC in methanol is presumably due to some aggregation by solubility issue. The similarity of the fluorescence quantum yields in methanol confirms that the photophysical properties of **1**, which can be affected by intermolecular behaviours, are less dependent of its concentration. Considering that at higher concentration of **1** in methanol, its fluorescence quantum yield corresponds to its less aggregated state, in aqueous solution the significantly increased fluorescence quantum yield above the CMC point is due to the micelle formation of **1**. The formation of **1** into a self-assembled micellar structure and the comparatively high fluorescence quantum yield of the CM give rise to an efficient fluorescence sensing signal.

### Amplified fluorescence quenching studies

**Figure 3:**
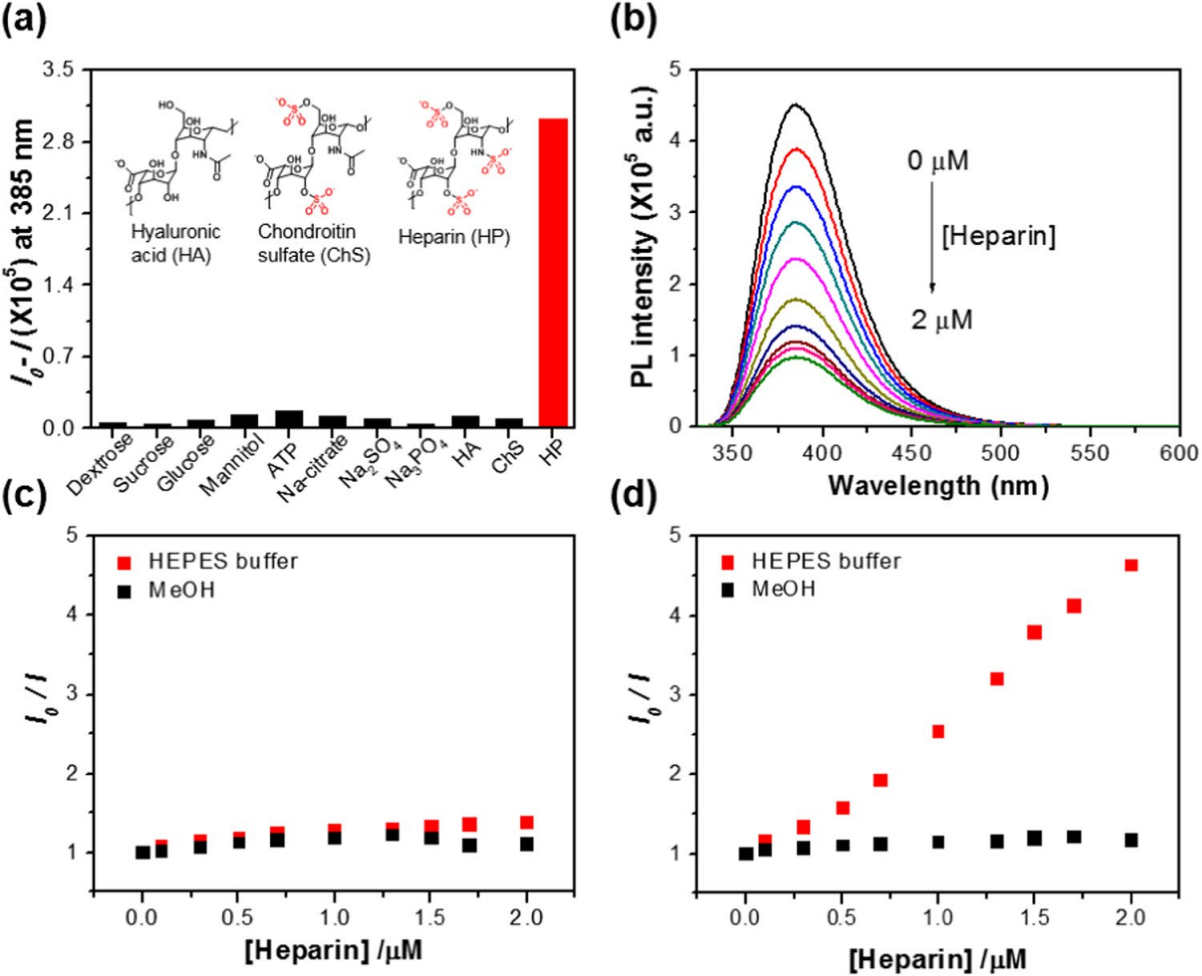
Efficient fluoregenic detection of heparin (HP) *via* signal amplification of 1. (**a**) fluorescence intensity changes of **1** (5.0 × 10^−6^ M) upon the addition of various analytes (1.7 × 10^−6^ M) and (**b**) various amounts of HP (0 ~ 2.0 × 10^−6^ M) in 10 mM HEPES buffer solution at pH 7.4. excitation was at 320 nm and fluorescence intensity was monitored at 385 nm. (**c**) Stern-Volmer (SV) plot below (1.0 × 10^−6^ M) and (**d**) above (5.0 ×10^−6^ M) the CMC of **1** in MeOH and in 10 mM HEPES buffer solution at pH 7.4.

We then evaluated the electron transport properties between the aggregated fluorophores in the CM, by looking for evidence of amplified fluorescence quenching with various selected analytes: dextrose, sucrose, glucose, mannitol, ATP, sodium citrate, Na_2_SO_4_, Na_3_PO_4_, sodium hyaluronate (HA), chondroitin sulfate sodium salt (ChS), and HP sodium salt (HP) (Fig. 3a) in 10 mM HEPES buffer solution, pH 7.4 at 25 °C. The results show that the micellized **1** (5.0 × 10^−6^) M has high selectivity for HP *via* fluorescence suppression, where the maximum quenching reaches approximately 78.4% when 2.0 × 10^−6^ M of HP was incubated (Fig. 3b). It is also observed that the fluorescence changes of micellized **1** induced by the HP are independent of the presence or absence of other analytes, indicating the very good selectivity toward HP in aqueous solution (Supplementary Fig. 9). This high selectivity is presumably due to the strong propensity of the cationic surface of micelles to electrostatically interact with the HP that has a relatively higher density of negative charges than other analytes. As shown in Supplementary Fig. 10, the fluorescence quenching results using PSS (polystyrene sulfonic acid) with high charge density in aqueous media support this hypothesis, where the polymeric PSS efficiently quenched the fluorescence emission of micellized **1** rather than its monomeric SDS (sodium dodecyl sulfate). The HP complexation of micellized **1** was also studied by SEM, AFM, XRD, and DLS analyses as shown in Supplementary Figs. 11 and 12. The consistent feature is that the size of micellized **1** increased approximately three- to four-times by the HP complexation in SEM, XRD, and DLS results. Interestingly, it was observed in the SEM image that micelles were gathered together, suggesting that micelle particles are gathered around the HP with opposite charges. As shown in Supplementary Fig. 11c, the XRD profile of the micelle shows the characteristic crystalline peaks. The first significant peak observed at 2θ = 5.88° appeared from the 1.5 nm spacing, which may indicate periodic array distance of micellized **1**. Even after the addition of the HP, the crystallinity is intact. As the inter-micellar aggregation and retained crystallinity were observed in the SEM image and XRD, respectively, it is likely that the micellar structures are not changed and gathered around HP while upon the addition of HP.

This fluorescence quenching of **1** was further investigated by Stern-Volmer (SV)^25^ quenching experiment in methanol and HEPES buffer solution at pH 7.4. The changes in the fluorescence spectrum are shown in Fig. 3b and Supplementary Fig. 13, and the resulting SV plot obtained by monitoring the fluorescence intensity of **1** at λ = 385 nm is shown in Fig. 3c,d and Supplementary Fig. 14. The corresponding SV quenching constants are also summarized in Supplementary Table 2. Interestingly, the SV plots for HP quenching of **1** (5.0 × 10^−6^ M) above the CMC point display different features in methanol and aqueous solution. In aqueous solution, the SV plot displays an upward profile with SV quenching constant of *K*_SV_ = 1.3 × 10^6^ M^−1^ with increasing HP concentrations. This large *K*_SV_ value suggests that the fluorescence quenching is static and presumably due to photoinduced electron transfer from aggregated coumarin units to sulfonate functional groups of HP^26,27^. By contrast, below the CMC point of **1** (1.0 × 10^−6^ M), the fluorescence quenching efficiency is not significant with low *K*_SV_ value (*K*_SV_ = 2.3 × 10^4^ M^−1^). The analytical detection limit (ADL) of the micellized **1** (5.0 × 10^−6^ M) is less than approximately 8.4 × 10^−9^ M, which is 224-fold greater than that below the CMC point. In methanol, as expected, the fluorescence of **1** is not efficiently quenched with much lower gradient (*K*_SV_ = 4.6 × 10^2^ M^−1^ for [**1**] = 1.0 × 10^−6^ M and *K*_SV_ = 4.1 × 10^3^ M^−1^ for [**1**] = 5.0 × 10^−6^ M) regardless of its concentration, and its ADL is comparable to that below the CMC point in the aqueous solution^28^. Overall, the results show that above the CMC point in aqueous solution, micellized **1** can selectively and sensitively sense HP and report on it with an amplified fluorescence quenching, suggesting that a singlet exciton is facilitated in the agglomerated state of coumarin in micelles, as in a molecular wire system^29,30^.

**Figure 4:**
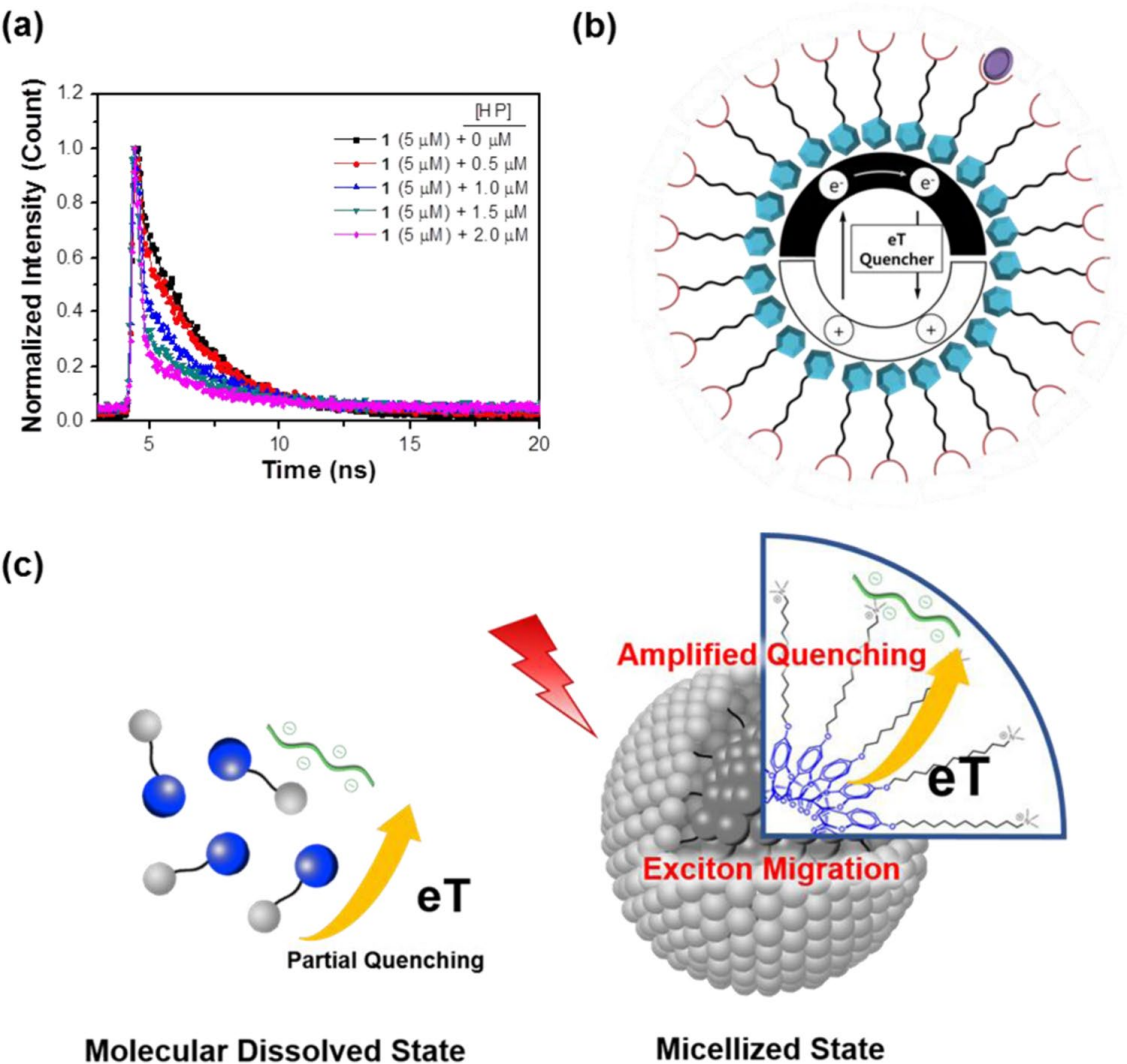
Amplified fluorescence quenching. (**a**) time-resolved spectroscopic study above (5.0 × 10^−6^ M) the CMC of **1** titrated with HP in 10 mM HEPES buffer solution at pH 7.4. (**b**) quenching mechanism of the “molecular agglomerate” effect in conjugated micelles (CM) for analytes. (**c**) schematic illustration for amplified fluorescence quenching of micellized **1**.

The micelle-induced sensing behaviour of **1** for HP was further elucidated *via* TCSPC in methanol and HEPES buffer solution at pH 7.4. Figure 4a shows the fluorescence decays of micellized **1** (5.0 × 10^−6^ M) detected over 390 nm in the HP concentration range of 0 to 2.0 × 10^−6^ M. The fluorescence lifetimes (*τ*_*i*_) and their relative amplitude contributions (RA%) to the overall decays are summarized in Supplementary Table 3. Importantly, the fluorescence decays rapidly with increasing HP concentration, where the long-lived decay kinetics of micellized **1** (5.0 × 10^−6^ M) shows decreased contribution for HP complexation, while the amplitude of the short-lived component is relatively increased, i.e., the average lifetime (*τ*_av._^**1**-HP^ = 0.78 ns/[HP] = 2 μM) is about 3.1 times faster than that of micellized **1** itself (*τ*_av_^1^ = 2.40 ns). This evidences that the efficient fluorescence quenching is dominated by interfluorophore singlet exciton delocalization and rapid intermolecular exciton transport due to aggregation of fluorophores in the micellized system^31,32^.

Taken together, the steady-state and time-resolved fluorescence experiments described above clearly demonstrate that the fluorescence quenching is amplified in the CM. Considering that unmicellized or less micellized state of **1** showed moderate fluorescence quenching effect in both methanol and aqueous solution, a micellar structure with the hydrophobic π-extended conjugated system in the inner part provides a channel for efficient energy and electron transport. Figure 4b illustrates how amplified quenching is processed in the CM. The excited electron (a bound electron-hole) is generated on the aggregated fluorophore upon the absorption of light, and moves very rapidly along the π-extended aggregated backbone that acts as a conduit for exciton. The fluorescence is quenched when the exciton is close to monomeric units occupied with the quencher (HP). The highly efficient exciton migration in the excited state results in quenching of many monomeric units in the CM, amplifying the quenching effect for the bound analytes (Fig. 4c).

### Application of sensors in serum

**Figure 5:**
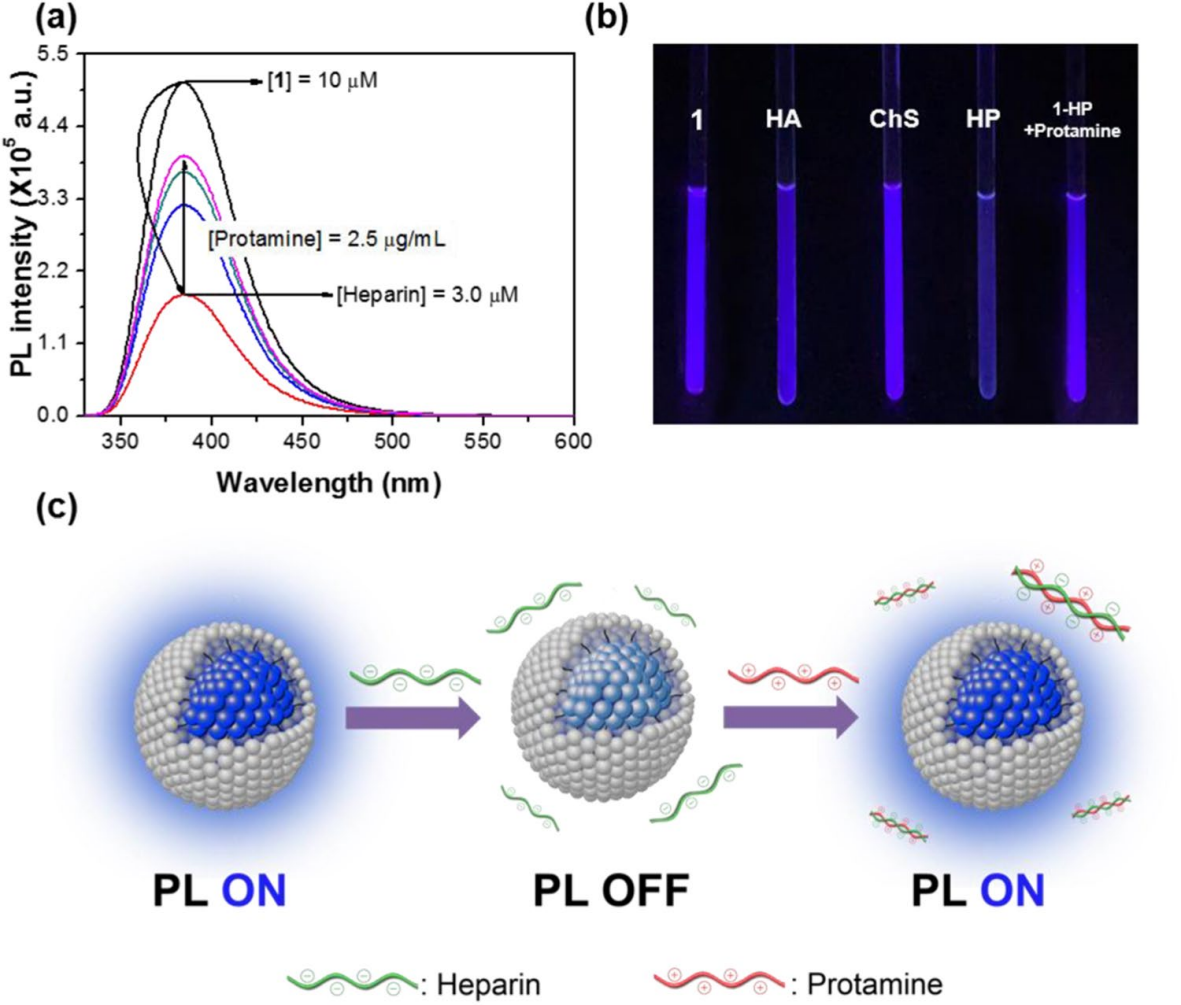
Fluorescence turn-on assay for trace detection of protamine. (**a**) fluorescence turn-on assay based on **1**-HP complex upon the addition of various amounts of protamine in 10% diluted serum (10 mM HEPES buffer/serum, 9:1, *v/v*) at pH 7.4. [**1**] = 1.0 × 10^−5^ M and [HP] = 3.0 × 10^−6^ M. (**b**) colour photographs for fluorescence response of micellized **1** for HA, ChS, and HP, and **1**-HP for protamine. [**1**] = 1.0 × 10^−5^ M, [HP] = 3.0 × 10^−6^ M. [ChS] = 3.0 × 10^−6^ M, [HP] = 3.0 × 10^−6^ M, and [protamine] = 2.5 × 10^−6^ g/mL. (**c**) sensing mechanism for the heparin (HP) detection of the conjugated micelle (CM).

In order to provide a practical insight into the biosensor applications of micellized **1**, the detection of HP and protamine was investigated in serum at room temperature (Fig. 5). The solution for HP assay was prepared with 5.0 × 10^−6^ M of **1** in 10% diluted serum (10 mM HEPES buffer/serum, 9:1, *v/v*) at pH 7.4. and incubated for 10 minutes before addition of the analyte. Figure 5a and Supplementary Fig. 15a illustrate the fluorescence quenching at various HP concentrations in 10% diluted serum, where the fluorescence intensity has gradually decreased with increasing HP concentration. Considering that the normal concentration range of HP in 10% diluted serum is 0.17 to 1.0 μM, the micellized **1** allows detection of abnormal HP concentration in practical serum conditions^16,33^. Interestingly, despite the difference in analytical conditions for pure water, buffer solution, and diluted serum, the SV quenching constants are comparable (Supplementary Fig. 16 and Supplementary Table 2). This suggests that the amplified quenching effect of micellized **1** by HP does not require specific environmental conditions to induce efficient exciton migration, but does require the micelle formation and ion pairing between the CM and analytes^32^. As expected, micellized **1** shows no significant effect on other competitive biomaterials, including HA and ChS in 10% diluted serum (Supplementary Fig. 15b). The images of its fluorescence colour changes also clearly show the high selectivity to HP (Fig. 5b). The addition of small amounts of protamine reverses this quenching as shown in Fig. 5a,b. Once HP has been stripped away by the protamine, the analyte protein prevents further association of the CM with HP, resulting in the recovery of the quenched fluorescence (Fig. 5c). The system, i.e., micellized **1** and HP complex, where the quenchers act as transducers for the turn-on sensory response, can also provide a good sensing platform for high sensitivity.

## Conclusions

We have devised a new, micelle-based sensing platform, where the amphiphilic building block **1** displays self-assembly featuring the hydrophobic π-extended conjugated system in the inner part and hydrophilic functionality at the periphery in aqueous media. The exciton efficiently migrates through aggregated fluorophores in a CM, thereby amplifying the fluorescence quenching (*K*_SV_ > 1.2 × 10^6^ M^−1^) as in a conjugated polymeric system. Based on the superior photophysical properties of the micellized conjugated system, comprised of efficient signal transductions and high fluorescence quantum yield, this approach has potential use for enhancing the sensitivity of the previously developed, single-molecule sensors.

## Experimental

### Synthesis

7-Hydroxy-4-methylcoumarin was purchased from Sigma-Aldrich and used as received. Precursor **2** and amphiphilic building block **1** were prepared in a good yield using the synthetic methods described in the literature^34,35^.

#### Synthesis of precursor 2

To a solution of 7-hydroxy-4-methylcoumarin (1.0 g, 5.67 mmol) in dried CH_3_CN (30 mL), anhydrous K_2_CO_3_ (1.18 g, 8.51 mmol) was added. After stirring for 30 min, 1,12-dibromododacane (2.05 g, 6.24 mmol) was added to the reaction mixture. The resulting mixture was vigorously stirred at 80 °C for 6 hours under argon gas. After the reaction mixture was cooled to room temperature, the solvent was removed *in vacuo*. The reaction mixture was extracted with CH_2_Cl_2_ (200 mL). The organic layer was separated and washed with water (100 mL) and dried over anhydrous MgSO_4_, and the solvent was evaporated to yield a white solid. The pure product was isolated by column chromatography on silica gel using ethyl acetate:hexane (1:5,*v/v*) as the eluent. Yield: 75%; mp 53.4 °C; ^1^H NMR (500 MHz, CDCl_3_): 7.49 (d, 1 H), 6.86, 6.84 (dd, 1 H), 6.81 (d, 1 H), 6.13 (s, 1 H), 4.01 (t, 2 H), 3.41 (t, 2 H), 2.39 (d, 3 H), 1.88–1.78 (dq, 4 H), 1.49–1.28 (m, 16 H); ^13^C NMR (100 MHz, CDCl_3_): ^13^C NMR (500 MHz, CDCl_3_): 162.42, 161.55, 155.48, 152.77, 125.64, 113.57, 112.85, 111.98, 101.50, 68.79, 34.27, 33.01, 29.60, 29.51, 29.16, 28.94, 28.35, 26.13, 18.87.

#### Synthesis of 1

To a solution of **2** (1.00 g, 2.36 mmol) in dried tetrahydrofuran (THF; 15 mL), trimethylamine/THF (2.36 mmol) was added. The resulting mixture was vigorously stirred at room temperature for 24 hours under argon gas. After the reaction mixture was cooled to room temperature, the solvent was removed *in vacuo*. The reaction mixture was extracted with CH_2_Cl_2_ (200 mL). The organic layer was separated and washed with water (100 mL) and dried over anhydrous MgSO_4_, and the solvent was evaporated to yield a white solid. The white precipitate was collected by vacuum filtration and washed with THF (500 mL). Yield: 96.5%; mp 166.6 °C; ^1^H NMR (500 MHz, DMSO-*d*_6_): 7.68 (d, 1 H), 6.96 (d, 1 H), 6.20 (s, 1 H), 4.08 (t, 2 H), 3.28 (t, 2 H), 2.40 (d, 3 H), 1.74–1.64 (dq, 4 H), 1.43–1.24 (m, 16 H); ^13^C NMR (100 MHz, DMSO-d^6^): 161.78, 160.19, 154.77, 153.47, 126.47, 113.00, 112.43, 111.05, 101.13, 68.24, 52.13, 28.98, 28.93, 28.80, 28.72, 28.51 28.42, 25.75, 25.41 22.03, 18.14; ESI-MS: Calculated for C_25_H_40_NO_3_^+^, *m/z* (M^+^) 402.3003, Observed for *m/z* (M^+^) 402.3004.

### Sample preparation

A 1 cm quartz cuvette was used for all spectral measurements. Stock solution (10.0 mM) of **1** was prepared in DMSO. The solution was kept at room temperature for one hour before use. HP sodium salt from porcine intestinal mucosa, chondroitin sulfate sodium salt, sodium hyaluronate, dextrose, sucurose, glucose, mannitol, sodium citrate, sodium sulfate and ATP (stock solutions = 10.0 mM in H_2_O) were tested to evaluate the metal ion binding properties of **1**. To obtain clear TEM, SEM and AFM images, micellized **1** was filtered using a hydrophilic syringe filter (0.45 μm) to remove large particles. The films of amphiphilic building block **1** were prepared by drop-casting a filtered solution of micellized **1** onto copper grid (for TEM) or silicon wafer (for SEM and AFM). Then, the films were sufficiently washed in a deionized water bath and dried at room temperature for 24 hours to remove residual water. To obtain PXRD patterns for micellized **1**, the sample was prepared by filtration of a very small powder formed by putting a perfectly dissolved **1** (70 mg) in DMSO (0.5 mL) into the water (3 mL). Micellized **1**-HP complexed sample was also prepared by filtration of a very small powder by adding HP (40 mg) to the aqueous solution (0.5 mL) of micellized **1** (70 mg).

### Serum lipid extraction with organic solvent

Normal human serum purchased from Sigma-Aldrich chemical company was used and its specifications are listed in Supplementary Table 4. Extraction with a chloroform-methanol mixture was based on the methodology of Folch *et al*.^36^ After 4 mL of normal human serum was added to 10 mL of chloroform-methanol (2:1, *v/v*), the mixture was agitated manually for 1 minute and centrifuged at 4000 rpm for 10 minutes at room temperature. After centrifugation, the aqueous phase was collected and repeated once more with hexane^37^.

### Characterization of amphiphilic building block 1

NMR spectra were recorded using a Bruker Ascend (AVANCE III 500), operated at 500 MHz for ^1^H-NMR and a Bruker Ascend (AVANCE III 400), operated at 100 MHz for ^13^C-NMR. HRMS was measured by Synapt G2-HDMS mass spectrometer (Waters). UV/Vis absorption spectra were recorded using an Analytikjena (SPECORD 200) and Sinco Mega-2100 UV-Vis spectrometer. Steady-state fluorescence spectra were obtained with a Shimadzu fluorometer RF-6000. Lifetime measurements were carried out using a PicoQuant FluoTime 200 Fluorescence Lifetime Spectrometer. TEM images were acquired with JEOL 1230 TEM operated at 100 kV. Atomic force microscope (AFM) images were obtained with a Nanoscope IIIa. Scanning Electron Microscopy (SEM) images were obtained with a Field Emission Scanning Electron Microscope (FE-SEM). The X-ray powder diffraction patterns were recorded from 3° to 60° with an interval of 0.02° on a Rigaku Miniflex 600 equipped with a Cu sealed tube (λ = 1.5406 Å). Dynamic light scattering (DLS) experiments were performed with Zetasizer Nano S90 from Malvern.

## Supplementary information

Supplementary information.
