# Supplementary material for: Micellization-induced amplified fluorescence response for highly sensitive detection of heparin in serum
Source: Sci Rep. 2020 Jun 10;10:9438. doi: 10.1038/s41598-020-66360-8 (PMC7287074; doi:10.1038/s41598-020-66360-8)
Supplement: Supplementary file 1 — Supplementary information. [file 41598_2020_66360_MOESM1_ESM.docx]

**Supplementary Information**

For

Micellization-induced amplified fluorescence response for highly sensitive detection of heparin in serum

Yeon Jin Jang^1,2,a^, Boyun Kim^1,2,a^, Euijin Rho^3^, Hyunuk Kim^3^ & Seoung Ho Lee^1,2^*

*^1^Department of Chemistry, Daegu University, Gyeongsan 38453, Republic of Korea,*

*^2^Institute of Natural Sciences, Daegu University, Gyeongsan 38453, Republic of Korea*

*^3^Institute Energy Materials Laboratory, Korea Institute of Energy Research, 152 Gajeong-ro, Yuseong-gu, Daejeon, 34129, Republic of Korea*

Experimental page S3

Supplementary Figure 1 page S3

Supplementary Figure 2 page S4

Supplementary Figure 3 page S4

Supplementary Figure 4 page S5

Supplementary Figure 5 page S6

Supplementary Figure 6 page S6

Supplementary Figure 7 page S7

Supplementary Table 1 page S8

Supplementary Figure 8 page S9

Supplementary Figure 9 page S9

Supplementary Figure 10 page S10

Supplementary Figure 11 page S11

Supplementary Figure 12 page S11

Supplementary Figure 13 page S12

Supplementary Figure 14 page S12

Supplementary Table 2 page S13

Supplementary Table 3 page S13

Supplementary Figure 15 page S14

Supplementary Figure 16 page S14

Supplementary Table 4 page S15

APPENDIX page S16

**Experimental**

**Materials.** All chemicals used in the synthesis were of reagent grade and used without further purification. 7-Hydroxy-4-methylcoumarin and normal human serum were purchased from Sigma-Aldrich Chemical Company. 1,12-dibromoundecane, trimethylamine/THF(13%/*wt*) were purchased from Tokyo Chemical Industry Company. Acetonitrile were purchased Alfa Aesar. Tetrahydrofuran, dimethyl sulfoxide were bought from Daejung Company. Silica gel (Merck, 230-400 mesh) was used for chromatographic purification of all of intermediate and target molecules. All other chemicals and solvents were purchased from Sigma-Aldrich, Fisher Scientific, or Acros Chemical Company and used as received.


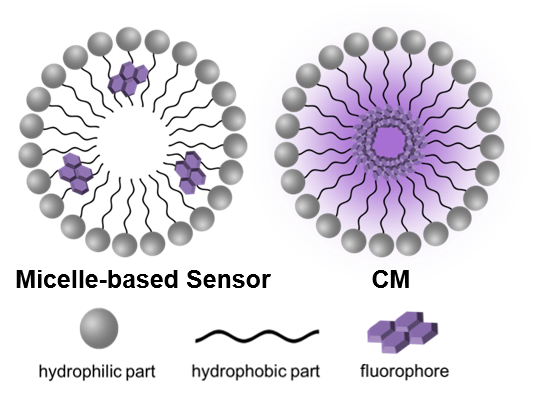


**Supplementary Figure 1.** Schematic illustration of two different kinds of micelle-based fluorescent sensing platforms.


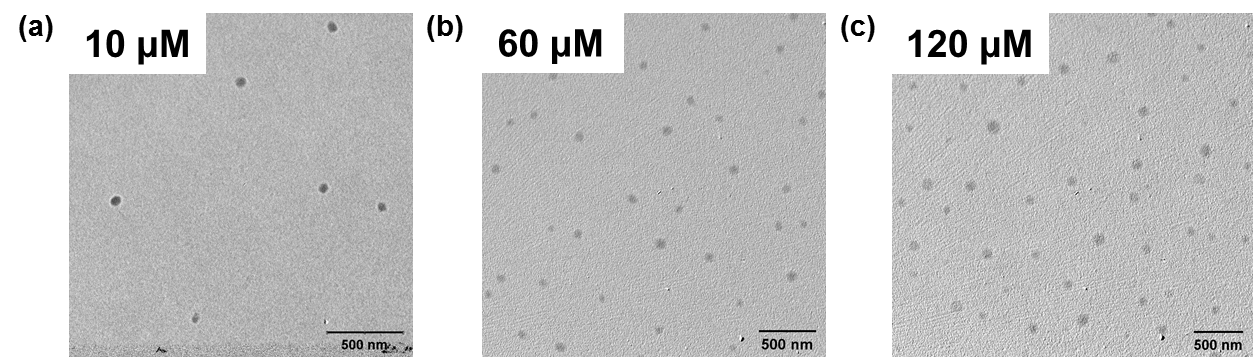


**Supplementary Figure 2.** HAADF-STEM images (scale bars, 500 nm).


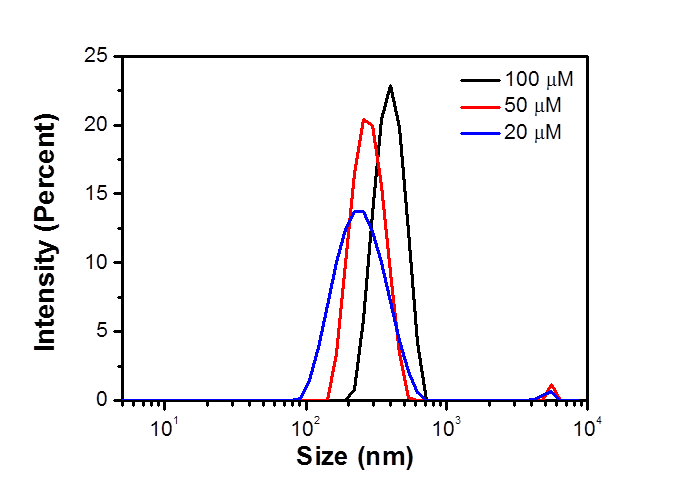


**Supplementary Figure 3.** Volume-based distribution of the hydrodynamic diameter obtained from dynamic light scattering (DLS) of **1** with increasing its concentration in H_2_O.


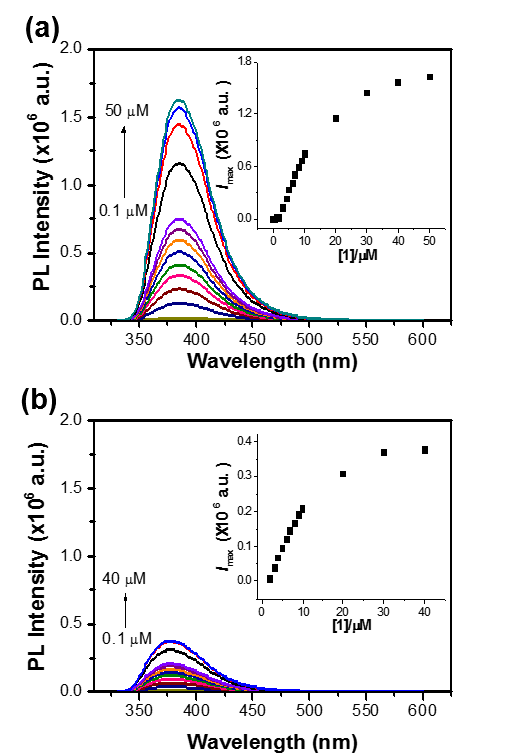


**Supplementary Figure 4.** Fluorescence intensity changes of **1** with increasing concentration in (a) H_2_O and (b) MeOH. Excitation at 320 nm.

**
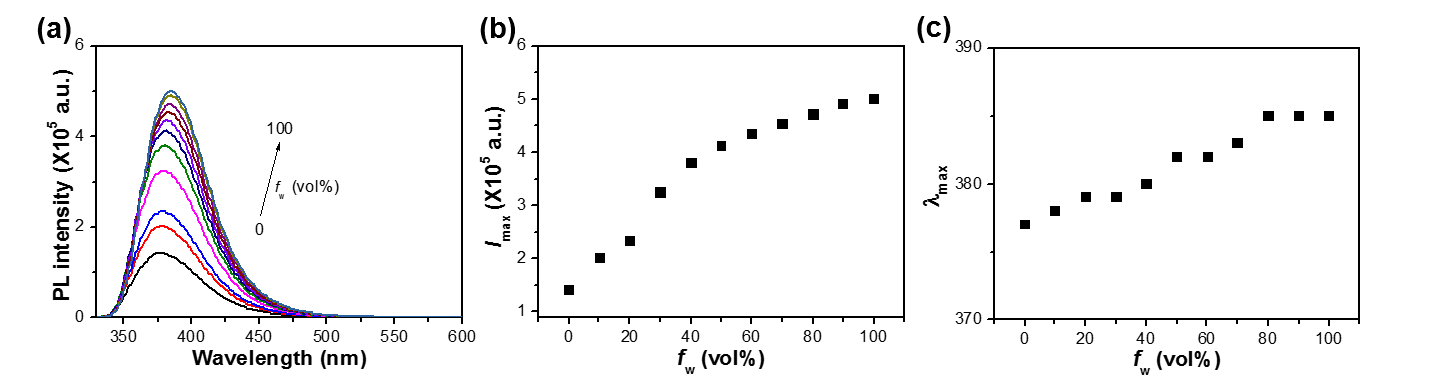
**

**Supplementary Figure 5.** (a) Fluorescence intensity change of **1** (5.0 × 10^-6^ M) in MeOH/H_2_O with increasing H_2_O fraction; (b) plot of fluorescence intensity change at λ_max_ of **1** (5.0 × 10^-6^ M) in MeOH/H_2_O with increasing H_2_O fraction; (c) plot of fluorescence wavelength change at λ_max_ of **1** (5.0 × 10^-6^ M) in MeOH/H_2_O mixture with increasing H_2_O fraction.

**
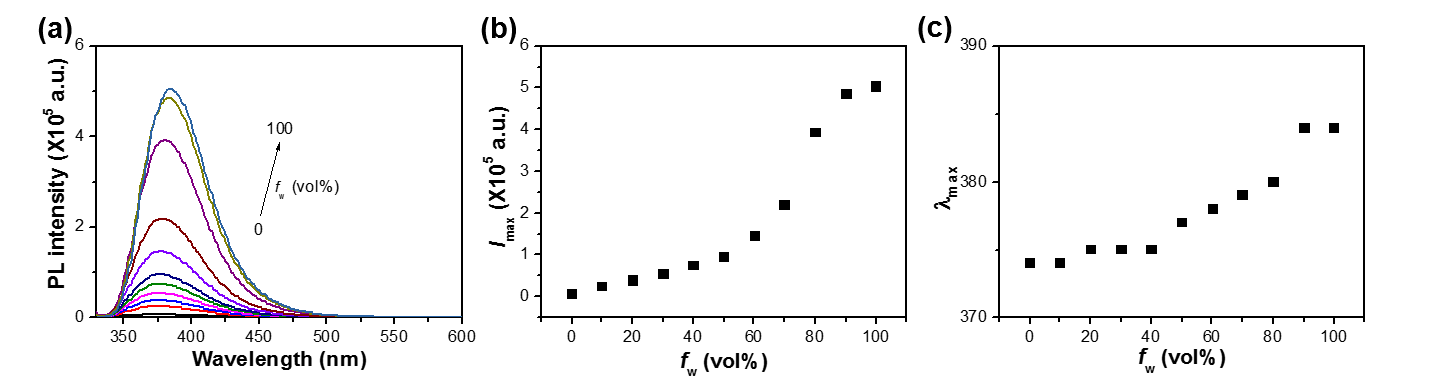
**

**Supplementary Figure 6.** (a) Fluorescence intensity change of **1** (5.0 × 10^-6^ M) in THF/H_2_O with increasing H_2_O fraction; (b) plot of fluorescence intensity change at λ_max_ of **1** (5.0 × 10^-6^ M) in THF/H_2_O with increasing H_2_O fraction; (c) plot of fluorescence wavelength change at λ_max_ of **1** (5.0 × 10^-6^ M) in THF/H_2_O mixture with increasing H_2_O fraction.


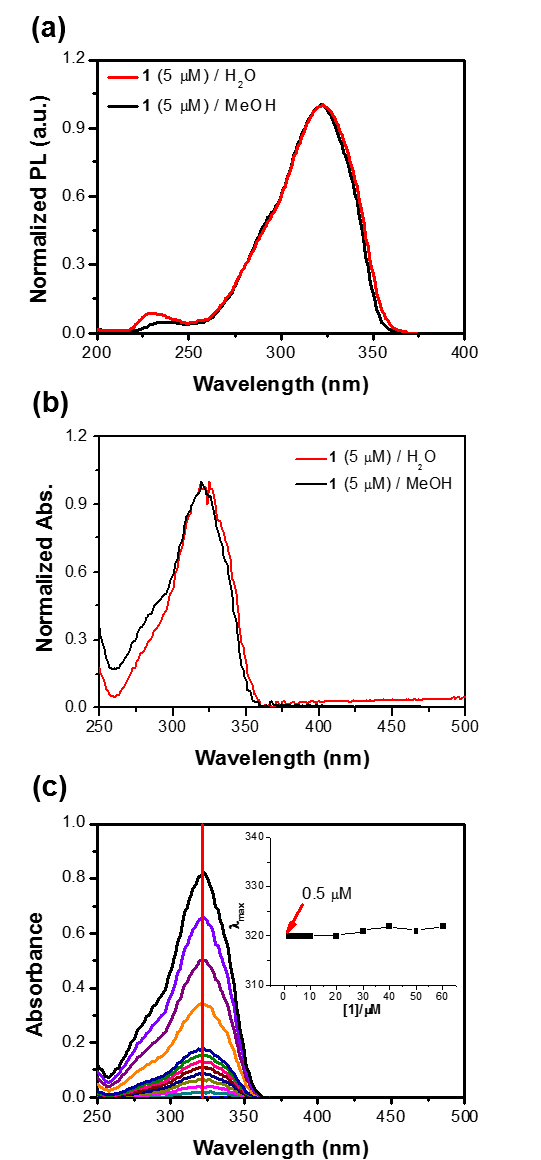


**Supplementary Figure 7.** (a) Florescence excitation spectra of **1** (5.0 × 10^-6^ M) in MeOH and H_2_O. Excitation at 320 nm; (b) UV/Vis absorbance spectra of **1** (5.0 × 10^-6^ M) in MeOH and H_2_O; (c) UV/Vis absorption changes of **1** with increasing concentration in H_2_O.

**Supplementary Table 1.** Fluorescence lifetimes (*τ*_i_, ns) and relative amplitudes (RA, %) for **1** in MeOH and 10 mM HEPES buffer solution at pH 7.4.

|  | Compd. **1** | | *τ*_av._ (ns) | Short-lived decay | | Long-lived decay | | χ^2^ |
| --- | --- | --- | --- | --- | --- | --- | --- | --- |
|  |  |  |  | *τ*_1_ (ns) | RA (%) | *τ*_2_ (ns) | RA (%) |  |
| H_2_O | | 1 μM | 1.96 | 0.25 | 25 | 2.53 | 75 | 0.99 |
|  |  | 5 μM | 2.40 | 0.22 | 3 | 2.45 | 97 | 0.99 |
|  |  | 10 μM | 2.39 | - | - | 2.39 | 100 | 0.99 |
| MeOH | | 1 μM | 0.21 | 0.21 | 100 | - | - | 0.98 |
|  |  | 5 μM | 0.33 | 0.26 | 95 | 1.65 | 5 | 0.99 |
|  |  | 10 μM | 0.40 | 0.29 | 91 | 1.83 | 9 | 0.98 |
|  |  | 20 μM | 0.43 | 0.29 | 85 | 1.25 | 14 | 0.98 |
|  |  | 30 μM | 0.58 | 0.36 | 73 | 1.17 | 27 | 0.99 |


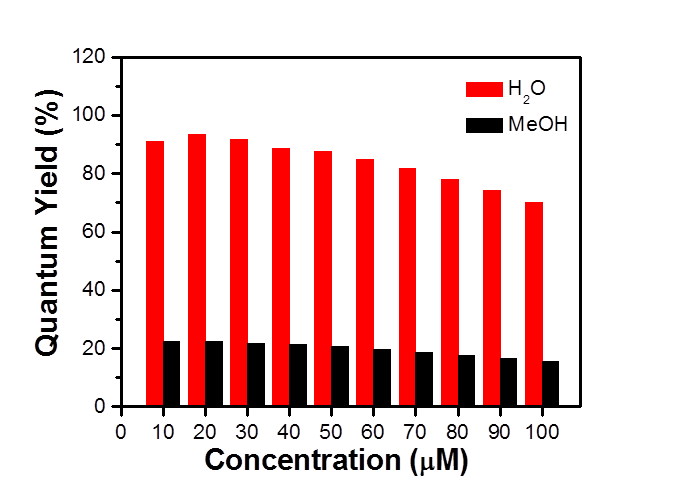


**Supplementary Figure 8.** Fluorescence quantum yields of **1** with increasing concentration in H_2_O and MeOH; 9,10-diphenylanthracene in cyclohexane as the standard, Φ_FL_=0.95.


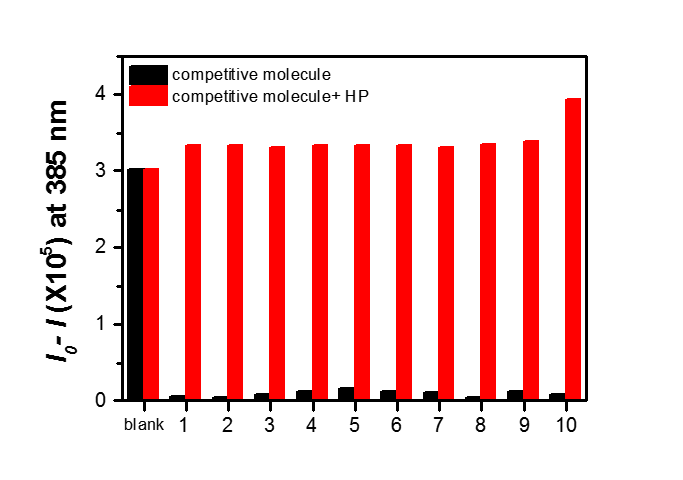


**Supplementary Figure 9.** Fluorescence intensity changes of **1** (5.0 × 10^-6^ M) in 10 mM HEPES buffer solution at pH 7.4 toward HP (1.7 × 10^-6^ M) in the presence of other analytes (1.7 × 10^-6^ M). 1: dextrose, 2: sucrose, 3: glucose, 4: mannitol, 5: ATP, 6: citrate, 7: Na_2_SO_4_, 8: Na_3_PO_4_, 9: HA, 10: ChS. Excitation at 320 nm; fluorescence intensity was monitored at 385 nm.


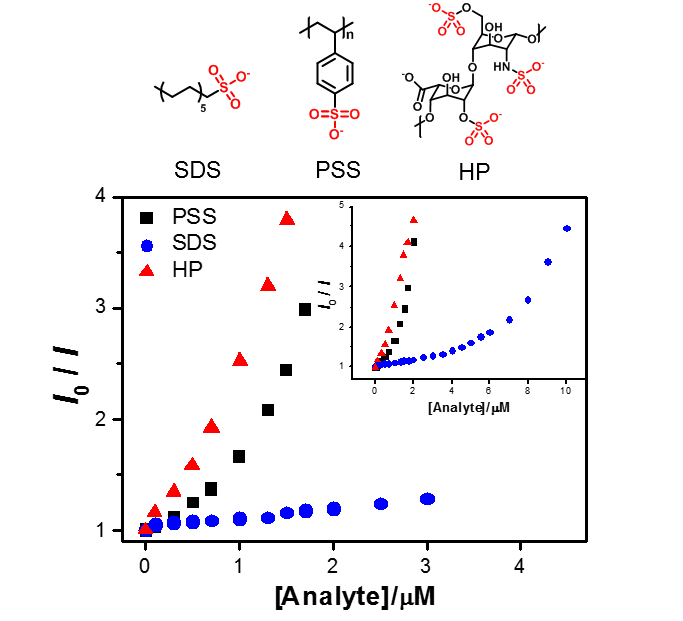


**Supplementary Figure 10.** Stern-Volmer plots of **1** (5 × 10^-6^ M) titrated with HP, PSS, and SDS in 10 mM HEPES buffer solution at pH 7.4; Excitation at 320 nm, fluorescence intensity was monitored at 385 nm.


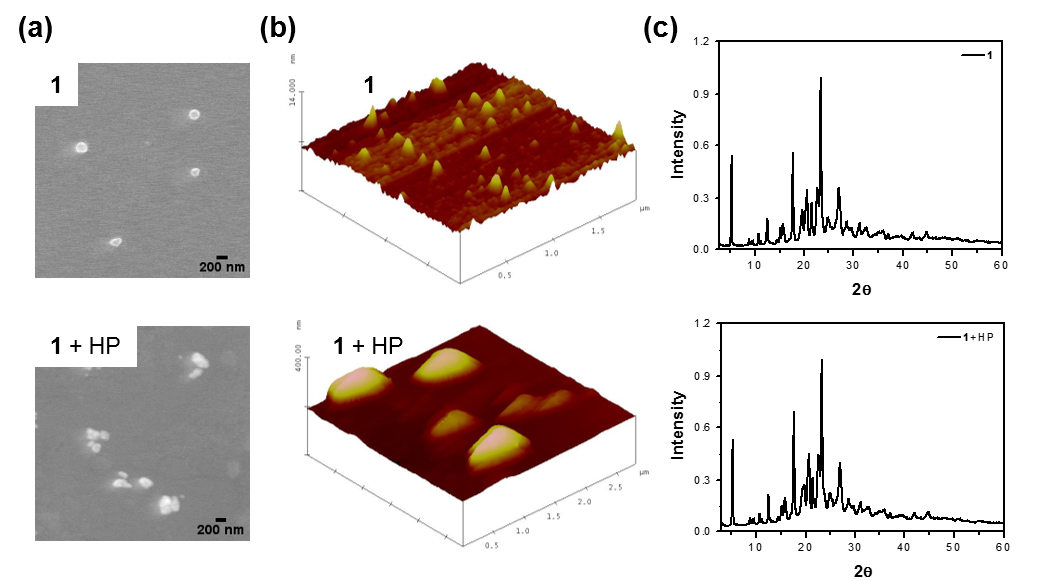


**Supplementary Figure 11.** (a) FE-SEM image, (b) AFM image, and (c) XRD patterns of **1** (1.0 × 10^-5^ M) upon the addition of HP (2.0 × 10^-6^ M).

**
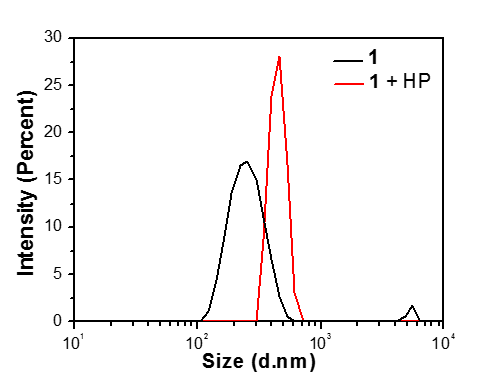
**

**Supplementary Figure 12.** Volume-based distribution of the hydrodynamic diameter obtained from dynamic light scattering (DLS) of **1** (1.0 × 10^-5^ M) upon the addition of HP (2.0 × 10^-6^ M).


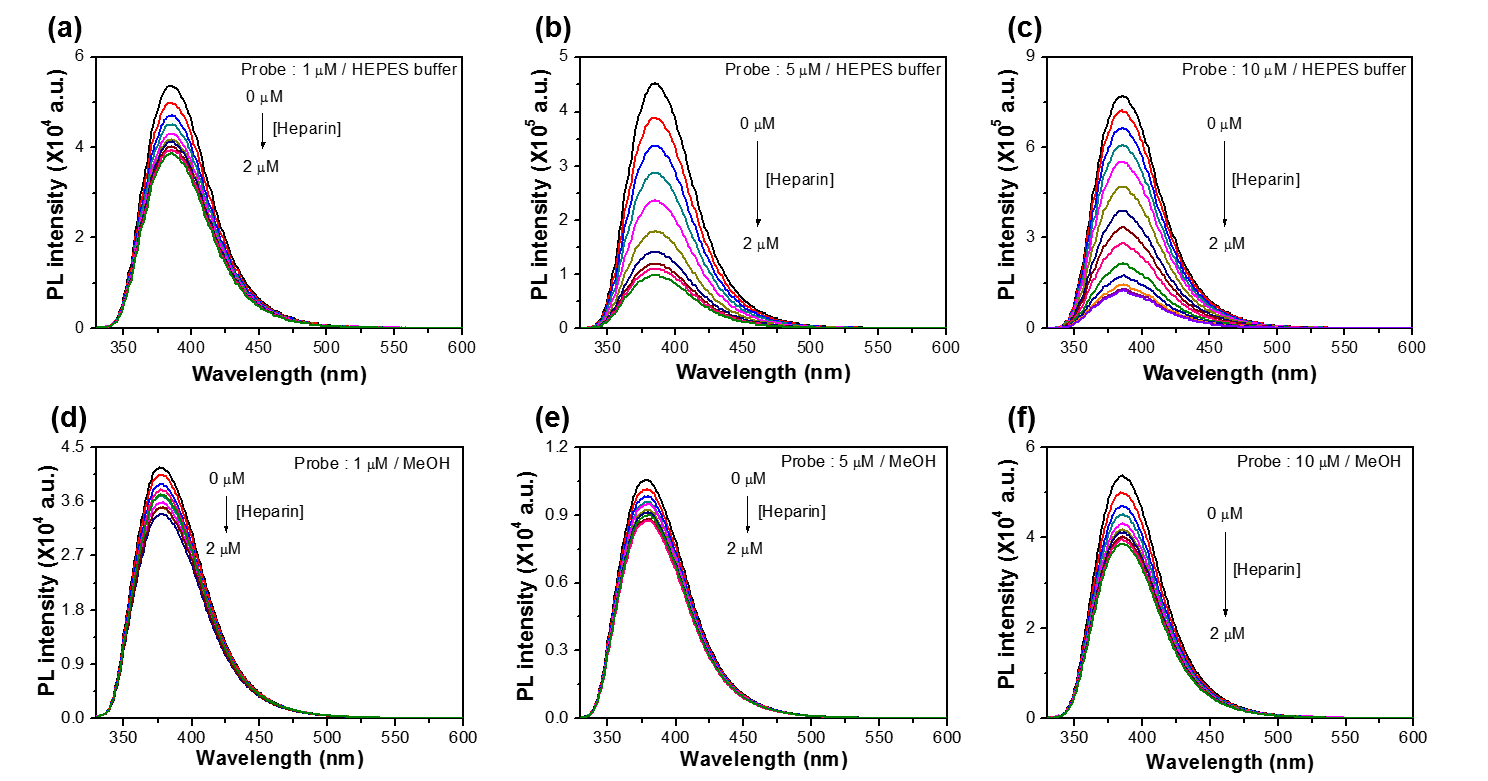


**Supplementary Figure 13.** Fluorescence intensity changes of **1** ((a) 1 × 10^-6^ M, (b) 5 × 10^-6^ M, and (c) 10 × 10^-6^ M) upon the addition of various amounts of heparin in 10 mM HEPES buffer solution at pH 7.4; Fluorescence intensity changes of **1**((d) 1 × 10^-6^ M, (e) 5 × 10^-6^ M, and (f) 10 × 10^-6^ M) upon the addition of various amounts of heparin in MeOH; Excitation at 320 nm.


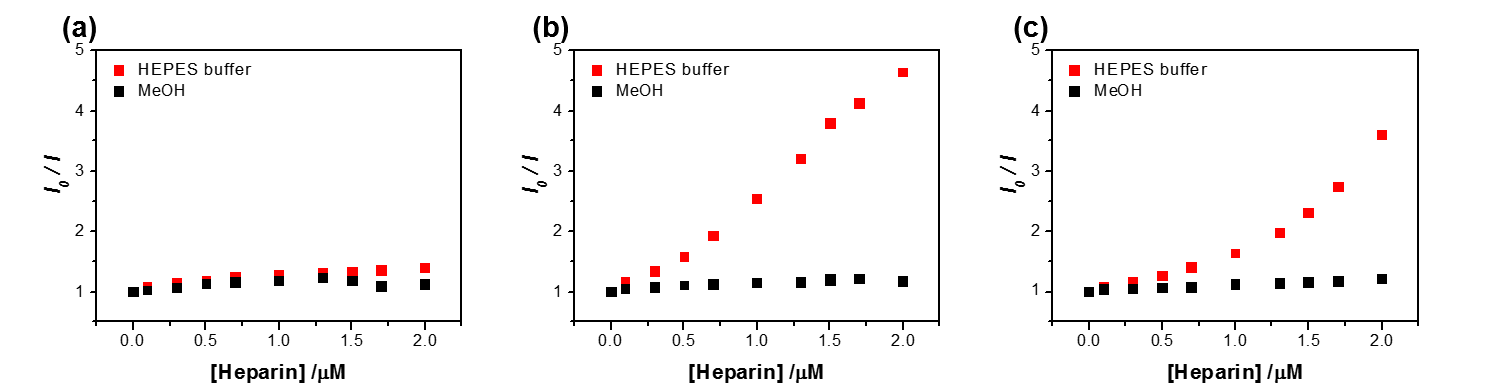


**Supplementary Figure 14.** Stern-Volmer plots of **1** ((a) 1 × 10^-6^ M, (b) 5 × 10^-6^ M, and (c) 10 × 10^-6^ M) titrated with heparin in 10 mM HEPES buffer solution at pH 7.4 and MeOH; Excitation at 320 nm, fluorescence intensity was monitored at 385 nm in HEPES buffer and 374 nm in MeOH, respectively.

| [Compd. **1]** | 1.0 🞨 10^-6^ M | 5.0 🞨 10^-6^ M | 1.0 🞨 10^-5^ M |
| --- | --- | --- | --- |
| SV constant | *K*_sv_ /M^-1^ | | |
| MeOH | 4.60 🞨 10^2^ | 4.10 🞨 10^3^ | 3.27 🞨 10^4^ |
| H_2_O | 2.28 🞨 10^5^ | 1.55 🞨 10^6^ | 7.23 🞨 10^5^ |
| 10 mM  HEPES buffer | 2.32 🞨 10^4^ | 1.25 🞨 10^6^ | 9.08 🞨 10^5^ |
| 10% serum | 1.52 🞨 10^3^ | 1.18 🞨 10^6^ | 8.2 🞨 10^5^ |

**Supplementary Table 2.** *K*_sv_*^a^* values for heparin quenching of **1** in MeOH, H_2_O, 10 mM HEPES buffer at pH 7.4, and 10% serum.

*^a^* Computed from linear fit at low quencher concentration.

**Supplementary Table 3.** Fluorescence lifetimes (*τ*_i_, ns) and relative amplitudes (RA, %) for **1** (5.0 🞨 10^-6^ M) upon the addition of heparin in 10 mM HEPES buffer solution at pH 7.4.

| [HP]/μM | τ_av._ (ns) | Short-lived decay | | Long-lived decay | | χ^2^ |
| --- | --- | --- | --- | --- | --- | --- |
|  |  | τ_1_ (ns) | RA (%) | τ_2_ (ns) | RA (%) |  |
| 0 | 2.40 | 0.22 | 3 | 2.45 | 97 | 0.99 |
| 0.5 | 2.35 | 0.23 | 4 | 2.44 | 95 | 0.99 |
| 1.0 | 1.92 | 0.22 | 23 | 2.44 | 77 | 0.99 |
| 1.5 | 1.84 | 0.24 | 67 | 2.61 | 67 | 0.98 |
| 2.0 | 0.78 | 0.30 | 82 | 2.97 | 17 | 0.97 |


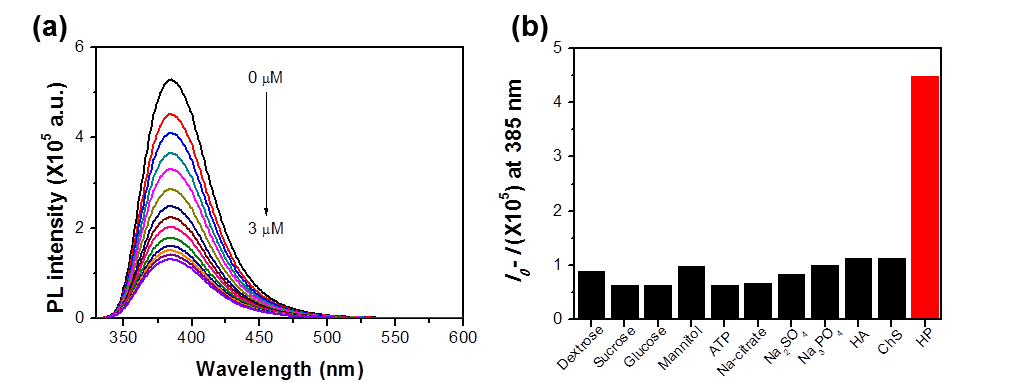


**Supplementary Figure 15.** Fluorescence intensity changes of **1** (5.0 × 10^-6^ M) upon the addition of (a) various amounts of heparin and (b) various analytes (10 × 10^-6^ M) in 10% diluted serum (10 mM HEPES buffer/serum, 9:1, *v/v*) at pH 7.4. Excitation was at 320 nm and fluorescence intensity was monitored at 385 nm.

**Supplementary Figure 16.** Stern-Volmer plots of **1** (5 × 10^-6^ M) titrated with heparin in H_2_O, 10 mM HEPES buffer, and 10% diluted serum (10 mM HEPES buffer/serum, 9:1, *v/v*); Excitation at 320 nm, fluorescence intensity was monitored at 385 nm in H_2_O, 10 mM HEPES buffer and 10% diluted serum, respectively.

**Supplementary Table 4.** Specification for normal human serum that was purchased from Sigma-Aldrich chemical company. Data was collected from Sigma-Aldrich chemical company sample information.

| TEST | Specification |
| --- | --- |
| Appearance (Color) | Colorless to Brown-Yellow to Brown |
| Appearance (Form) | Liquid |
| pH | 7.0 – 9.0 |
| Iron (UG%) | 40 – 100 |
| Source: Male donors  Within the United States | Conforms |
| Processing Country of Origin  United States | Conforms |
| Osmolality  Expressed in MOSM/KG H_2_O | 260 - 340 |
| Sterility by USP Guidelines | Pass |
| Hemoglobin | ≤ 25 mg/dl |
| Mycoplasma Test | None Detected |
| Endotoxin Level | ≤ 10 EU/ml |
| Cholesterol | 80 – 200 mg/dl |
| Triglyceride | 30 – 175 mg/dl |
| Glucose | 50 – 180 mg/dl |
| Sodium (Na) | 100 – 160 MEQ/L |
| Protein Content | 4.0 – 9.0 % |
| Tested For Infectious Agents | Tested |

All donor units are collected in donor centers located in the United States, which are licensed by the FDA.

**APPENDIX**


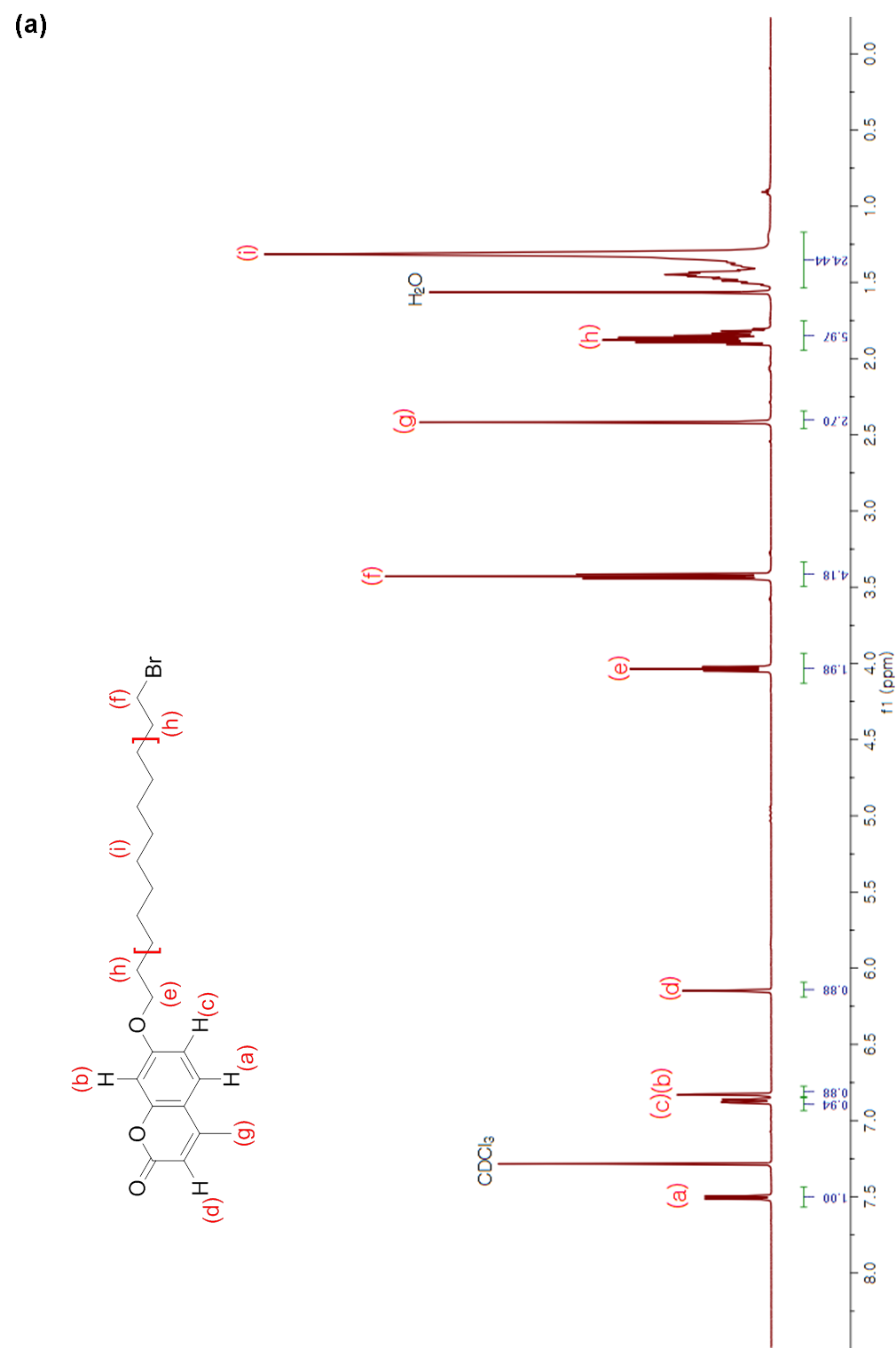


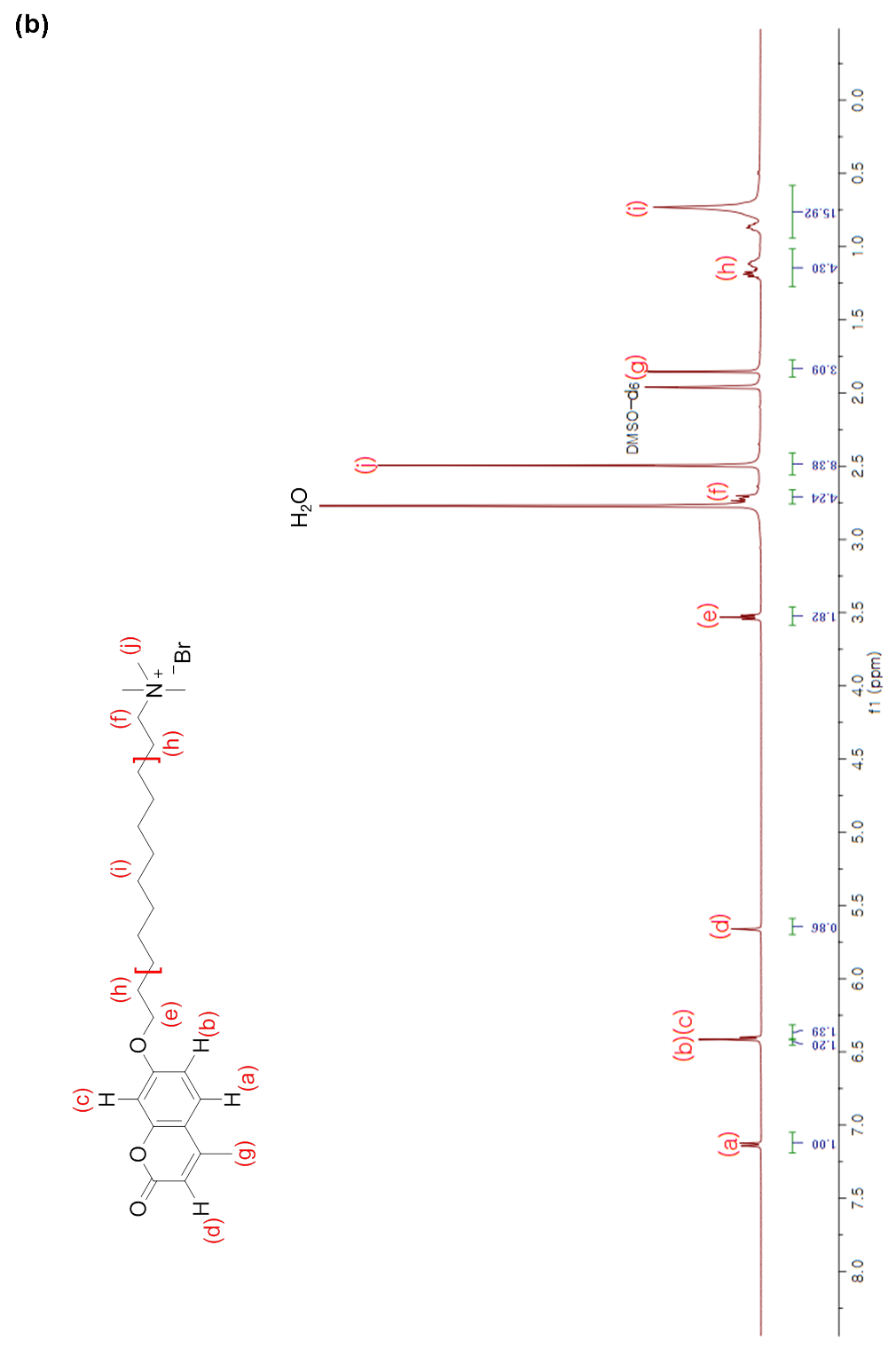


^1^H NMR spectra of (a) **2** in CDCl_3_ and (b) **1** in DMSO-*d_6_*.


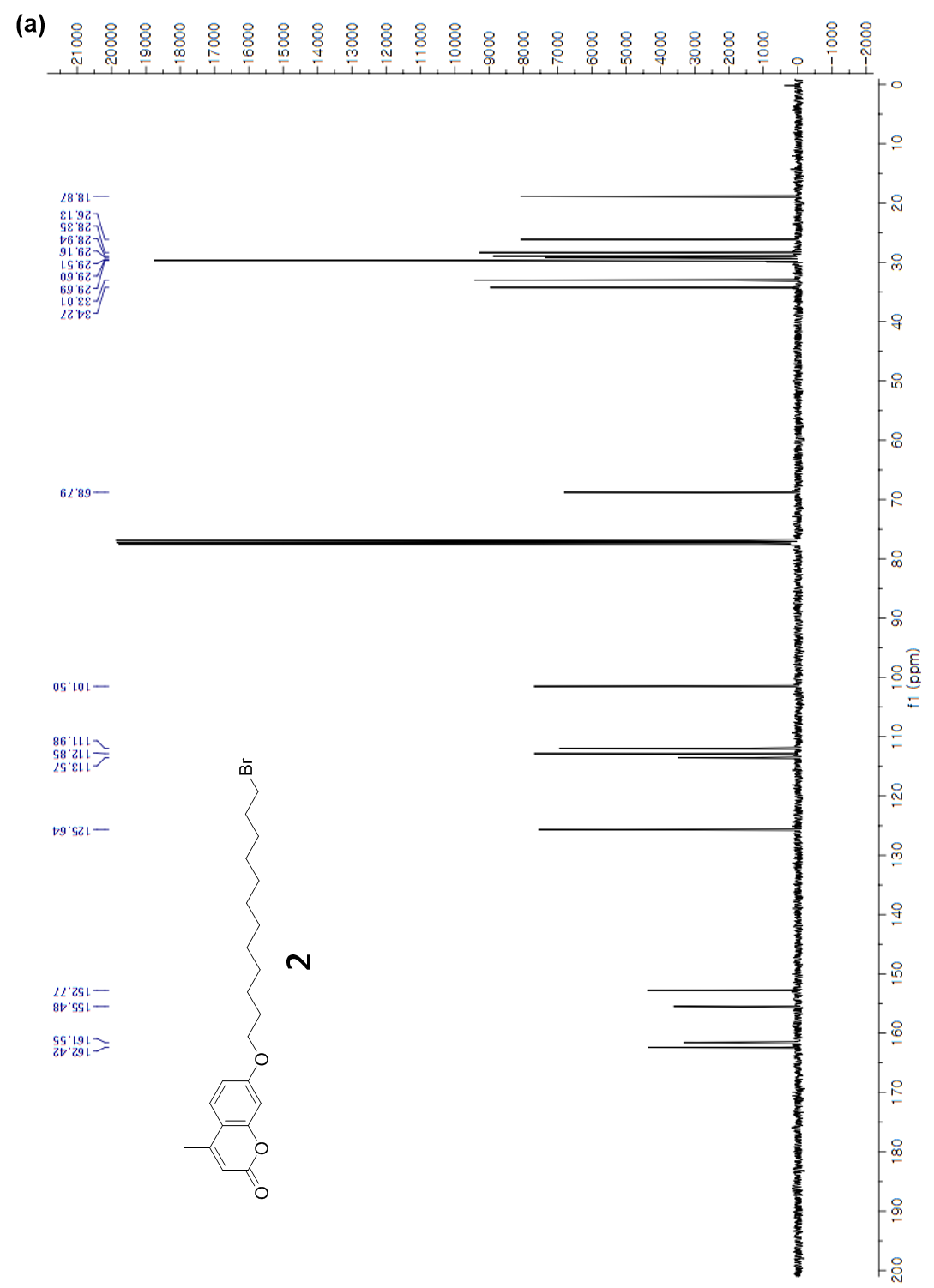


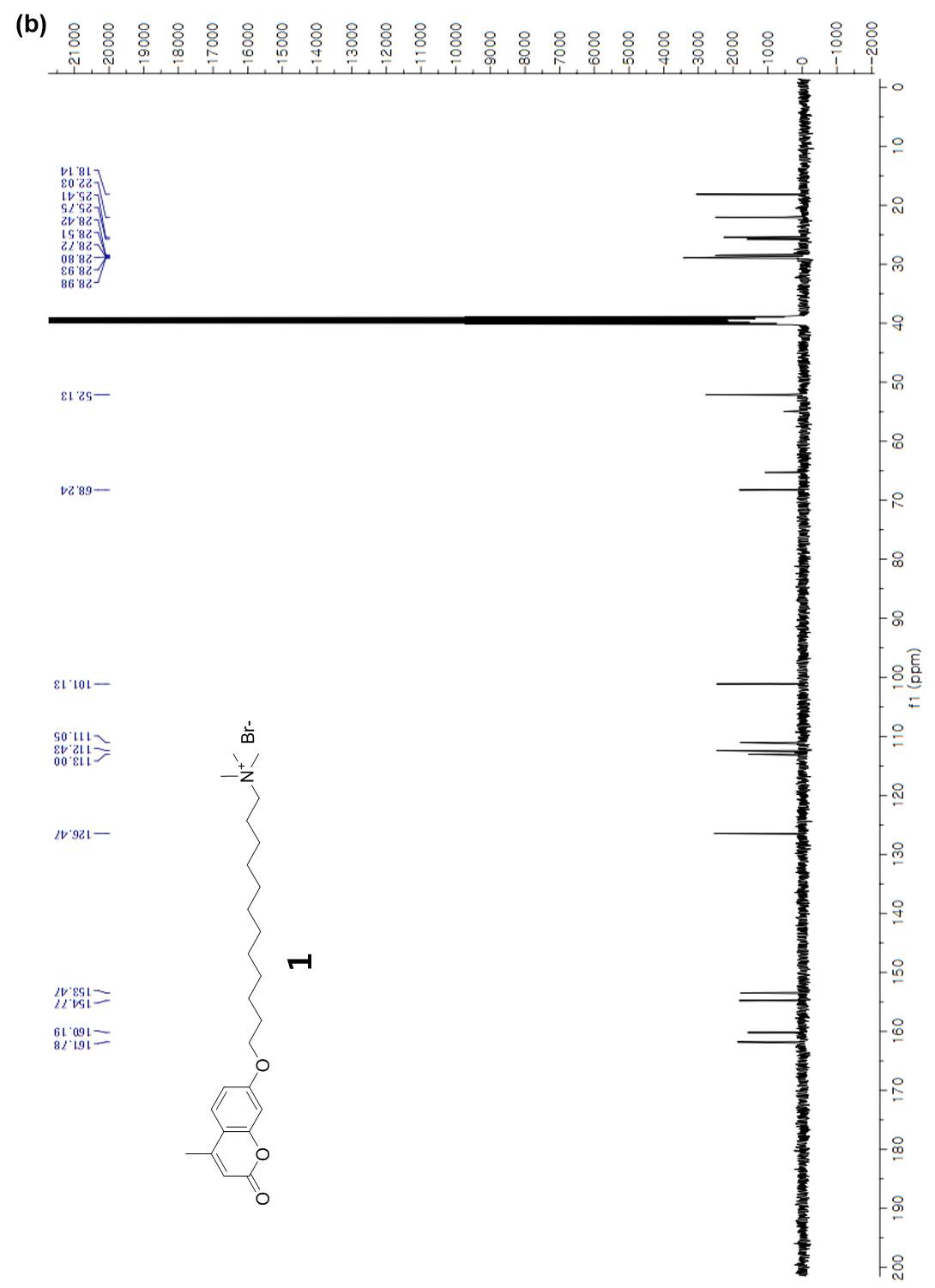


^13^C NMR spectra of (a) **2** in CDCl_3_ and (b) **1** in DMSO-*d_6_*.


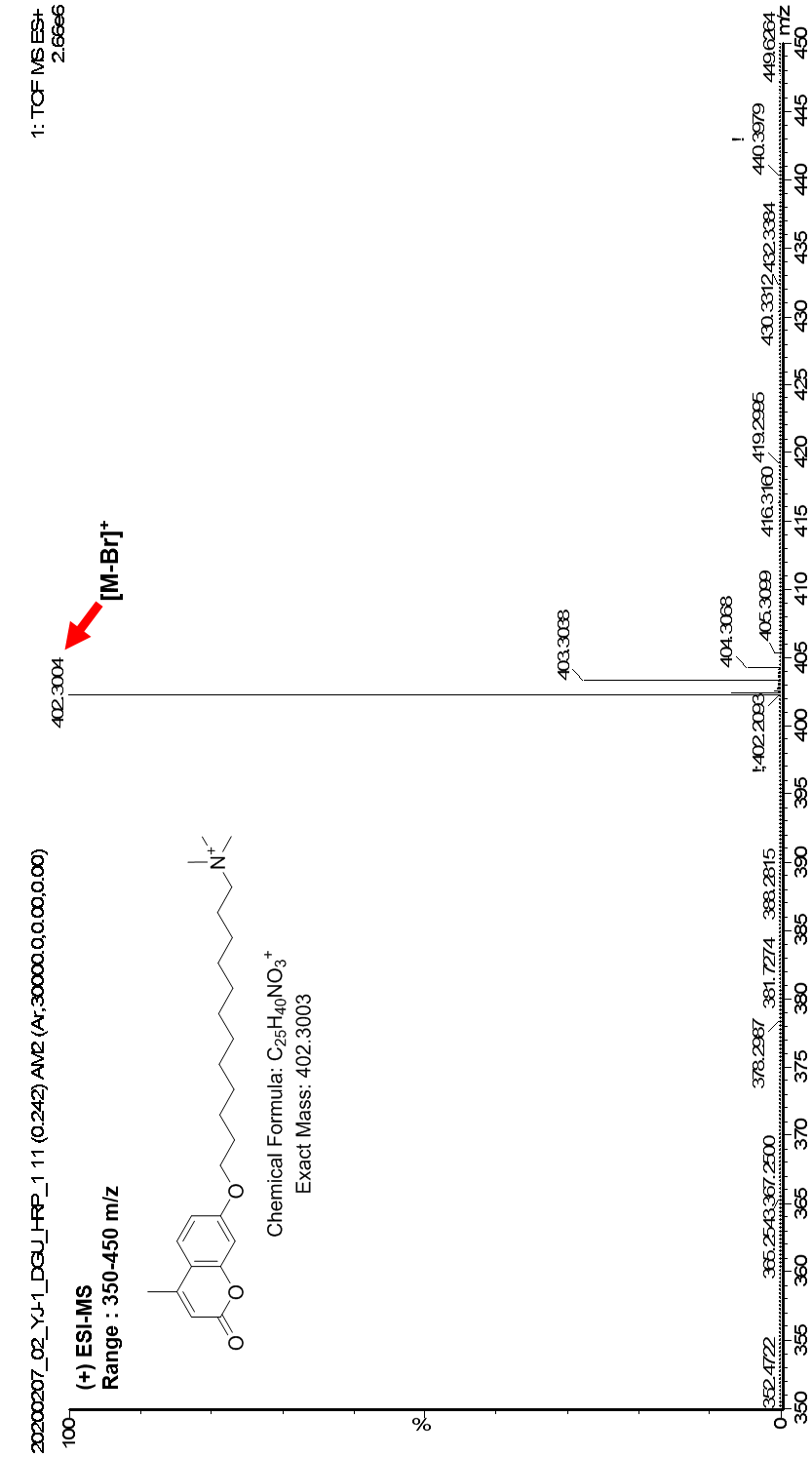


HRMS spectrum of **1**
